# Information needs for GPs on type 2 diabetes in Western countries: a systematic review

**DOI:** 10.3399/BJGP.2023.0531

**Published:** 2024-09-03

**Authors:** Tue Helms Andersen, Thomas Møller Marcussen, Ole Nørgaard

**Affiliations:** Danish Diabetes Knowledge Center, Copenhagen University Hospital – Steno Diabetes Center Copenhagen, Herlev, Denmark.; Danish Diabetes Knowledge Center, Copenhagen University Hospital – Steno Diabetes Center Copenhagen, Herlev, Denmark.; Danish Diabetes Knowledge Center, Copenhagen University Hospital – Steno Diabetes Center Copenhagen, Herlev, Denmark.

**Keywords:** general practice, primary health care, type 2 diabetes mellitus, information needs, knowledge needs, systematic review

## Abstract

**Background:**

Most people with type 2 diabetes receive treatment in primary care by GPs who are not specialised in diabetes. Thus, it is important to uncover the most essential information needs regarding type 2 diabetes in general practice.

**Aim:**

To identify information needs related to type 2 diabetes for GPs.

**Design and setting:**

Systematic review focused on literature relating to Western countries.

**Method:**

MEDLINE, Embase, PsycInfo and CINAHL were searched from inception to January 2024. Two researchers conducted the selection process, and citation searches were performed to identify any relevant articles missed by the database search. Quality appraisal was conducted with the Mixed Methods Appraisal Tool. Meaning units were coded individually, grouped into categories, and then studies were summarised within the context of these categories using narrative synthesis. An evidence map was created to highlight research gaps.

**Results:**

Thirty-nine included studies revealed eight main categories and 36 subcategories of information needs. Categories were organised into a comprehensive hierarchical model of information needs, suggesting ‘Knowledge of guidelines’ and ‘Reasons for referral’ as general information needs alongside more specific needs on ‘Medication’, ‘Management’, ‘Complications’, ‘Diagnosis’, ‘Risk factors’, and ‘Screening for diabetes’. The evidence map provides readers with the opportunity to explore the characteristics of the included studies in detail.

**Conclusion:**

This systematic review provides GPs, policymakers, and researchers with a hierarchical model of information and educational needs for GPs, and an evidence map showing gaps in the current literature. Information needs about clinical guidelines and reasons for referral to specialised care overlapped with needs for more specific information.

## Introduction

The International Diabetes Federation estimates that 537 million adults aged 20–79 years were living with diabetes in 2021; 90% had type 2 diabetes.^1^ People with type 2 diabetes (PWT2D) need lifelong management and support to maintain good health and avoid severe complications.^2^ Regular follow-up and an engaging patient–professional partnership are needed to enhance self-care activities.^3^ Nevertheless, only 23% of PWT2D reach composite treatment goals, including haemoglobin A1c, blood pressure, low-density lipoprotein cholesterol, and avoiding smoking.^4^ Furthermore, most care management programmes for PWT2D have limited effect on both metabolic and patient-centred outcomes.^5^

Most PWT2D are managed by GPs, who have limited time to treat PWT2D and address complications (in this article ‘GP’ is used to denote all physicians with daily responsibility for managing the condition in primary care, including family physicians, primary care physicians, and general internists). Patient encounters with GPs in the Western world last between 5 and 22 min,^6^ including routine examinations, self-management guidance, and questions. For every patient seen, clinicians had 0.57 questions, 51% of which they pursued; they found answers in 78% of the questions they pursued.^7^ Most questions pertain to treatment, followed by diagnosis and epidemiology.^7^^–^9 Despite the importance of searching for relevant literature, two-thirds of physicians found doing so unmanageable.^10^ Furthermore, GPs should be familiar with extensive information to treat various diseases in people of different ages, genders, and ethnicities.^11^^,^^12^

However, several systematic reviews have found that physicians do not seek the information they need because of information overload and lack of time, skills, and integration of health information.^7^^,^^8^^,^^13^^–^15 They most often turn to colleagues for information, along with easily accessible websites such as Google and Google Scholar, followed by journal articles and textbooks.^8^^,^^14^^,^^16^ Asking colleagues is considered an easy and informal method for gaining information. Yet, this information is likely to be opinion based and thus not a reliable evidence source.^17^

Physicians’ information needs generally pertain to prevention, screening, diagnosis, prognosis, therapy, and aetiology;^8^^,^^18^ however, to the best of the authors’ knowledge, no systematic review to date has assessed the specific information needed of GPs working with PWT2D, representing a significant literature gap. This systematic review therefore aims to identify information needs related to type 2 diabetes mellitus for GPs in Western countries.

**Table 1:** How this fits in

Most people with type 2 diabetes are treated by GPs who are not specialised in diabetes. This systematic review provides GPs, policymakers, educators, and researchers with a hierarchical model of information and educational needs for GPs and highlights important knowledge gaps. Furthermore, an evidence map illustrates substantial gaps in the literature.

## Method

The reporting of this systematic review followed the Preferred Reporting Items for Systematic reviews and Meta-Analysis (PRISMA) 2020 statement^19^ and the extension to the PRISMA Statement for reporting literature searches in systematic reviews (PRISMA-S).^20^

### Eligibility criteria

The population, interest, and context (PICo) framework was used to develop the research question, eligibility criteria, and search strategy.^21^ Records were eligible for inclusion if they:

- contained information on GPs (P);
- described information, knowledge, or educational needs related to type 2 diabetes (I); and
- pertained to primary care in Western countries (that is, in Europe, North America, or Oceania) (Co).

Records were excluded if:

- no primary data on GPs’ information needs were reported;
- the type of information needed was not specified;
- information needs were not self-reported;
- results specific to GPs were not provided;
- information needs were not disease related, for example, pertaining to patient beliefs or electronic systems; or
- sources were not journal articles.

### Search strategy

An electronic literature search for studies published from inception to 19 December 2022 was conducted in MEDLINE (Ovid), Embase (Ovid), PsycInfo (Ovid), and CINAHL (EBSCO). All searches were updated on 29 January 2024.

The search strategy was developed by an information specialist (the first author) and combined key concepts of GPs, information/knowledge needs, and type 2 diabetes mellitus. Concepts were searched using controlled vocabularies, for example, medical subject headings (MeSH), free-text words, and keywords when possible.

PubMed PubReMiner (version 1.31),^22^ the MeSH database, and Embase Emtree were explored to identify relevant controlled vocabulary terms and synonyms. The search strategy was developed in MEDLINE and translated to other databases using matching controlled vocabularies and database-specific syntax. The ability of the search string to identify 19 key articles was tested. Finally, a second information specialist (the last author) reviewed the search strategy. Supplementary Information S1 and Supplementary Table S1 contain the complete search strategy.

Backward and forward citation searches of included studies were conducted in citationchaser to identify relevant records not retrieved by the database search.^23^

### Reference management and deduplication

All records were uploaded to EPPI-Reviewer 6 (ER6),^24^ and duplicates with a similarity threshold of >0.9 were automatically removed. Groups of duplicates with a similarity threshold of <0.9 were manually checked, and remaining records were manually screened for duplicates not detected automatically. Records retrieved during the citation search were uploaded to ER6 and checked for duplicates internally and against all records found in the database search.

### Study selection and full-text retrieval

Pilot screening of 200 studies was performed to review and clarify eligibility criteria and increase the consistency of record screening. Two researchers then independently screened titles and abstracts of all records, resolving any disagreements through discussion and review by a third researcher. Full-text review of records used an identical process.

Full-text reports were retrieved electronically. If no electronic version was available, the authors first attempted to retrieve it from a research library and then emailed the first or corresponding author. The report was excluded if no answer was received within 1 month. Reports in languages other than those read by the authors (English, Danish, Swedish, and Norwegian) eligible by title and abstract were not included but are listed in Supplementary Information S2.

Information about data extraction and the form are available in Supplementary Information S3 and Supplementary Table S2.

### Quality appraisal

The Mixed Methods Appraisal Tool (MMAT, version 2018) was used for appraisal of heterogeneous studies.^25^ It comprises five questions for each type of study design (quantitative, qualitative, and mixed methods) and two questions pertaining to all studies. Response options are ‘yes’ (criterion met), ‘no’ (criterion not met), and ‘can’t tell’. MMAT guidelines suggest neither generating an overall score nor excluding studies based on appraisal results. Two researchers independently conducted quality appraisal, resolving any disagreements by discussion and, as needed, by a third researcher.

### Data synthesis

A narrative synthesis appropriate for systematically summarising studies with heterogeneous designs and identifying patterns within complex data was conducted. The purpose was explorative, using primarily a qualitative approach to integrate qualitative, quantitative, and mixed-methods studies, and comparison and contrast of findings to elucidate information needs.^26^

Initially, meaning units related to information needs were extracted verbatim from each included study. All authors then individually coded meaning units and grouped those related to the same subject into categories, for example, insulin initiation and insulin treatment were grouped under the insulin category.

All categories were discussed, and overarching themes were formed to create main categories, for example, insulin and pharmacological management were grouped into the main category of medication. Finally, codes and categories were reviewed and revised to ensure data were fully and transparently reported and captured identified information needs.

To understand gaps in existing evidence on the topic, an evidence map was also created.^27^

## Results

**Figure 1.**
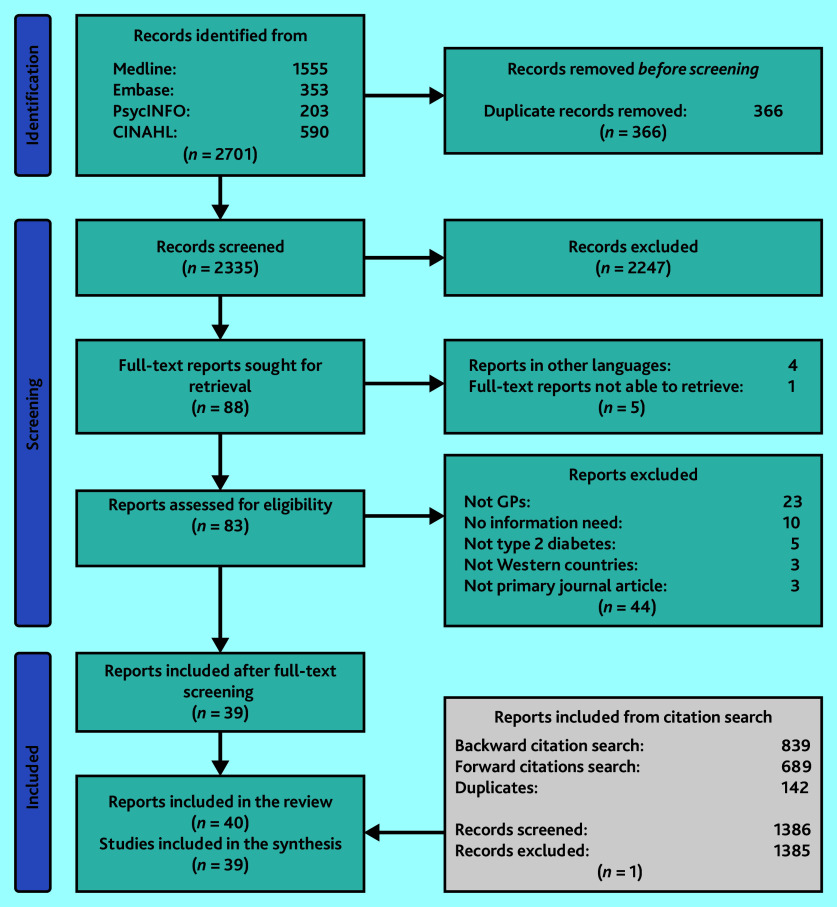
Flow diagram of study selection.

Database searches yielded 2701 records. Of these, 366 duplicates were excluded. Title and abstract screening excluded 2247 records. Of the remaining 88 records, 49 were excluded during full-text screening. Citation searches identified 1528 additional records, of which 142 were duplicates. A single study among the remaining 1386 records was included after screening. Thus, 40 reports from 39 studies were included in the review (Figure 1). Interrater reliability (IRR) was 83% for pilot screening and 92% for screening all records.

### Characteristics of studies

Overall, 39 studies published between 1990 and 2022 were included. More than two-thirds of included reports were published in or after 2013 (see Supplementary Figure S1). Eighteen studies were conducted in North America, 17 in Europe (including Turkey and Israel), and four in Oceania (see Supplementary Table S3 for study characteristics).

Twenty-nine included studies had descriptive quantitative designs, with data collected through questionnaires. Five studies had qualitative designs, with data collected by individual interviews. Five study designs were mixed methods, using descriptive questionnaires based on focus group responses and observations (see Supplementary Table S3).

Quantitative study response rates were 3.6%–91%. A total of 11 340 GPs participated in quantitative studies, including the questionnaire phase of the five mixed-methods studies. Qualitative studies (including the focus group phase of the mixed-methods studies) had a collective total of 133 participants.

None of the included studies provided all the information sought on participants (that is, gender, age, years of experience, number of PWT2D seen, and level of interest in diabetes). However, participants tended to be predominantly male, with a mean experience of >20 years (see Supplementary Table S3).

Eight studies adopted a broad study focus (see Supplementary Table S4), in which GPs were openly asked what information they needed. The remaining studies used various forms of testing researcher-defined knowledge gaps.

### Quality appraisal

Twenty-nine studies were appraised as quantitative descriptive. The risk of response bias was higher in 13 studies because of unclear or inadequate sampling strategies, insufficient information on statistical analyses, and unclear or insufficient representativeness of GPs. It was not possible to determine the risk of response bias in eight studies. Five studies were appraised as qualitative designs with a higher quality. Four had ‘yes’ responses to all appraisal questions, and it was not possible to determine the adequacy of data incorporation into findings in the fifth. Five studies were appraised as mixed-methods designs with a lower quality. Limitations derived from poor integration, description, or differentiation of methods; inadequate description of sampling strategies; low response rates and omission, or unclear derivation of qualitative findings (see Supplementary Table S5 for individual study appraisals).

The IRR agreement rate was 83% on all codes.

### Information needs synthesis

Findings about information needs are organised into eight main categories:

- medication;
- management;
- complications;
- diagnosis;
- risk factors;
- screening for diabetes;
- reasons for referral; and
- knowledge of guidelines.

#### Medication

Eighteen reports described the need for information on type 2 diabetes medications. Five addressed appropriate prescribing behaviour, pertaining to GPs’ ability to choose appropriate medications. Two studies by the same author found that GPs do not fully understand differences between glucagon-like peptide-1 (GLP-1) receptor agonists and dipeptidyl peptidase-4 inhibitors, including mechanisms of action and clinical indications.^28^^,^^29^ One study found that half of GPs could not correctly identify contraindications for metformin.^30^ Another found that GPs chose GLP-1 receptor agonists even when insulin was more appropriate.^31^ The fifth study found suboptimal use of angiotensin-converting enzyme (ACE) inhibitors and angiotensin II-receptor blockers, compared with sodium-glucose transport protein 2 (SGLT-2) inhibitors, in the treatment of comorbid chronic kidney disease (CKD) and type 2 diabetes.^32^

Four reports addressed non-specific pharmacological management of diabetes. Two included studies found that GPs wanted more education on oral diabetes medications.^33^^,^^34^ One study surveying GPs on knowledge about oral medications found that they most often correctly answered questions about metformin and sulfonylurea.^35^ One study identified a learning need among GPs on the use of injectable therapies.^36^

Twelve included studies addressed information needs related to insulin. Four studies reported findings related to initiating insulin treatment: one identified inadequate clinical experience as a barrier to insulin initiation,^37^ one reported on needed practical skills in injectable use,^31^ and two found that insufficient knowledge about insulin therapy was a barrier to initiation.^38^^,^^39^ One study found a specific lack of knowledge about needle sizes in injection devices.^40^ Two studies found that GPs requested education on adjusting and managing insulin treatment.^33^^,^^36^ Another two studies by the same author found a knowledge gap in navigating different insulin regimens,^28^^,^^29^ and three studies found a need for general knowledge about insulin characteristics.^34^^,^^35^^,^^41^

Nine studies addressed specific drug types. Two reported suboptimal use and knowledge of SGLT-2 inhibitors.^32^^,^^42^ In one study, GPs thought they knew enough about GLP-1 receptor agonists,^42^ but another study found that they were generally insufficiently aware of the effects of GLP-1 receptor agonists.^43^ De Lusignan *et al* also found that GPs prescribed GLP-1 receptor agonists when insulin was more appropriate.^31^ One study found that almost all responders knew the benefits of ACE inhibitors in delaying progression of diabetic nephropathy.^44^ One study found that GPs lacked knowledge on thiazolidinediones and their possible cardiovascular consequences^45^ to a significant degree.

#### Management

This category included all studies on management and treatment of type 2 diabetes that were unrelated to pharmacological treatment or management of complications. Nine reports contained findings related to managing type 2 diabetes. Four reported a lack of knowledge about hypoglycaemia, with Fisher *et al*^46^ directly linking insufficient knowledge to suboptimal treatment decisions.^33^^,^^39^^,^^46^^,^^47^ Fogelman *et al*^43^ and Phillips and Dromgoole^34^ found that 60% of GPs reported a lack of knowledge on nutritional issues and 68% reported an interest in more training.

Two studies found a need for knowledge about hypertension, and Rubin *et al* found that only 31% of GPs knew the blood pressure goal for PWT2D.^33^^,^^41^ Two studies found that GPs lacked knowledge about managing diabetes through fasting practices during Ramadan.^48^^,^^49^

Marsden and Grant analysed a broad range of topics that GPs requested education about.^33^ In addition to identifying knowledge gaps related to hypoglycaemia and hypertension, they found that managing weight problems (49% of GPs) and managing PWT2D at home (45% of GPs) were among the most common information needs related to type 2 diabetes.^33^

#### Complications

The complications category was the largest. Five articles reported a need for knowledge about managing complications. Phillips and Dromgoole reported that as many as 100% of GPs wanted education on vascular complications and 81% wanted information on acute complications.^34^ Cytryn *et al*^38^ and Thepwongsa *et al*^36^ found that they lacked confidence in managing comorbidities and complications. Shubrook *et al* reported that lack of knowledge of cardiovascular outcome trials had implications for care of PWT2D and cardiovascular disease.^50^ One study found that GPs overestimated the impact of strict metabolic control on macrovascular complications and overall mortality.^47^

Chu *et al*^32^ and Lo *et al*^51^ identified a need for education on prevention, earlier diagnosis, and early intervention for CKD. Lo *et al* also found that 73% of GPs were uncertain about the definition of CKD, and 80% wanted more information on managing patients with comorbid diabetes and CKD.^51^ One study found that GPs were unsure and sometimes factually incorrect when responding to questions about metformin use in patients with CKD.^30^

Two studies found that GPs expressed a need for education on eye complications of diabetes and specifically on retinal examination,^33^^,^^52^ with Delorme *et al* reporting GPs’ lack of confidence in their ability to screen for diabetic retinopathy.^52^ One study noted that GPs were well educated about diabetic eye disease.^53^

Chu *et al*^32^ and Wong *et al*^44^ reported that GPs used urine albumin-to-creatine ratio (uACR) screening insufficiently, and 39% chose inappropriate methods for detecting microalbuminuria. Two studies found that GPs lacked knowledge about the relationship between diabetes and periodontal diseases,^54^^,^^55^ as well as a general knowledge and training gap related to oral health.^55^

Just 2% of GPs requested training on diabetic foot complications^34^ but a different study found that they were dissatisfied with the accessibility and availability of diabetic foot ulcer guidelines.^56^

One study assessed knowledge of risk factors for CKD, reporting that most GPs correctly identified hypertension and diabetes mellitus as risk factors.^57^ Three studies investigated knowledge about specific complications, that is, Charcot neuroarthropathy, limited joint mobility (LJM), and non-alcoholic fatty liver disease (NAFLD). Bilello and Jupiter found inconsistent knowledge about managing Charcot neuroarthropathy among internal medicine physicians and GPs,^58^ and Alabdali *et al* found that most GPs who were asked were unaware that LJM was a diabetes complication.^59^ Lastly, Gracen *et al* found a gap in clinical practice related to the implementation of clear, evidence-based guidelines for NAFLD.^60^

#### Diagnosis

Three studies described the need for information on diagnosing type 2 diabetes. These focused on inadequate information about diagnostic criteria and cut-off points for tests such as fasting plasma glucose.^35^^,^^38^^,^^41^ Rubin *et al*^41^ found that only 49% of GPs knew the test criteria and Shahla *et al*^35^ found that they often incorrectly answered questions about the test criteria in the absence of type 2 diabetes symptoms.

#### Risk factors

Three studies analysed the need for knowledge about risk factors for diabetes. One asked GPs broadly about training needs, with 68% of responders indicating that they needed general knowledge about risk factors for type 2 diabetes.^34^ One study reported that GPs needed more training about obesity because they were not sufficiently informed on the risks and benefits of treatment, even though obesity is a well-known risk factor for type 2 diabetes.^61^ Rayanagoudar *et al* found a lack of knowledge among GPs about the long-term consequences of gestational diabetes, including the elevated risk of getting type 2 diabetes.^62^

#### Screening for diabetes

One report pertained to screening for type 2 diabetes. Whitford *et al* described the absence of knowledge on evidence related to screening for type 2 diabetes.^63^

#### Reasons for referral

Seven reports provided findings about information needs related to referring PWT2D to specialists. Three studies analysed the need for information on appropriate referral to a nephrologist,^32^^,^^44^^,^^51^ finding a need for more information and training on interpreting test results that should trigger referral, and on referral guidelines and pathways.

Two studies focused on type 2 diabetes and obesity, and potential reasons to refer patients for bariatric surgery.^61^^,^^64^ El-Beheiry *et al* found that nearly half of GPs did not feel adequately prepared to discuss the role of bariatric surgery and 75% did not follow referral criteria.^64^

One study evaluated information needs related to Charcot neuroarthropathy, concluding that lack of familiarity with symptoms may lead to fewer referrals than appropriate.^58^ The referral rate among GPs was 41.7%.

Finally, one study tested the tendency of GPs to refer patients to either endocrinologists or certified diabetes educators (CDE) under varying care circumstances.^28^ GPs were most likely to refer patients who were considering insulin pump therapy (>80%) or had experienced multiple hypoglycaemic events (50%). The most frequent reason for referring patients to CDEs was complex dietary issues.

#### Knowledge of guidelines

Eleven studies broadly indicated that GPs often lacked knowledge of appropriate guidelines, in terms of both familiarity with the most up-to-date guidelines and following guideline recommendations. Four studies found a general need for more information and training on applying type 2 diabetes guidelines.^10^^,^^28^^,^^65^^,^^66^ Williamson *et al* described GPs as less familiar with current guidelines, whereas Rätsep *et al* found inappropriate management of glycosylated haemoglobin and weight reduction when inquiring about guideline-recommended follow-up.^28^^,^^66^ Additionally, the use of appropriate medications was an issue.

Six reports provided findings about GPs’ limited knowledge of guidelines when treating type 2 diabetes and coexisting complications.^30^^,^^32^^,^^44^^,^^52^^,^^60^^,^^61^ Three studies on CKD, one on retinopathy, one on NAFLD, and one on obesity and bariatric surgery all found a lack of knowledge about the most appropriate guidelines for screening and managing comorbidities.

When asked about their preferences, 20% of GPs reported wanting more training on the organisation of diabetes care,^34^ an information need most often addressed by guidelines.

See Supplementary Table S4 for a full overview of information needs.

### Hierarchy of information needs

**Figure 2.**
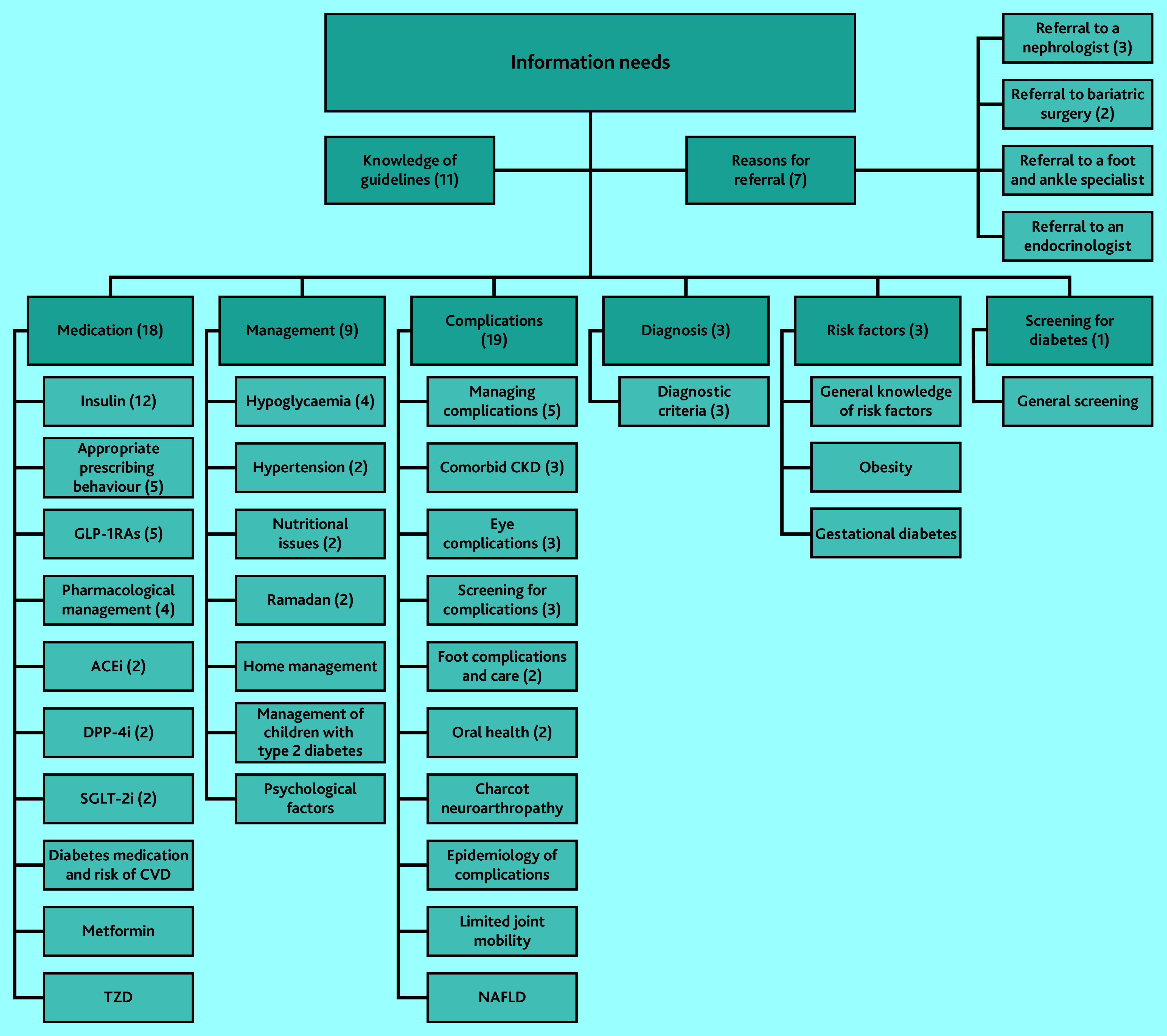
Overview of GPs’ information needs related to type 2 diabetes. Number of studies is in parentheses when there is more than one study. ACEi = angiotensin-converting enzyme inhibitor. CKD = chronic kidney disease. CVD = cardiovascular disease. DPP-4i = dipeptidyl peptidase-4 inhibitors. GLP-1RAs = glucagon-like peptide-1 receptor agonists. NAFLD = non-alcoholic fatty liver disease. SGLT-2i = sodium-glucose co-transporter-2 inhibitor. TZD = thiazolidinediones.

The included studies revealed information needs in eight main categories and 36 subcategories (Figure 2). Two main categories, knowledge of guidelines and reasons for referral, represent overarching themes relevant to all GPs. In practice, before addressing specific information needs in other categories, GPs should know the applicable guidelines and reasons for referral to more specialised care. Figure 2 thus depicts the hierarchy of both information needs and educational efforts for GPs.

### Evidence gaps

**Figure 3.**
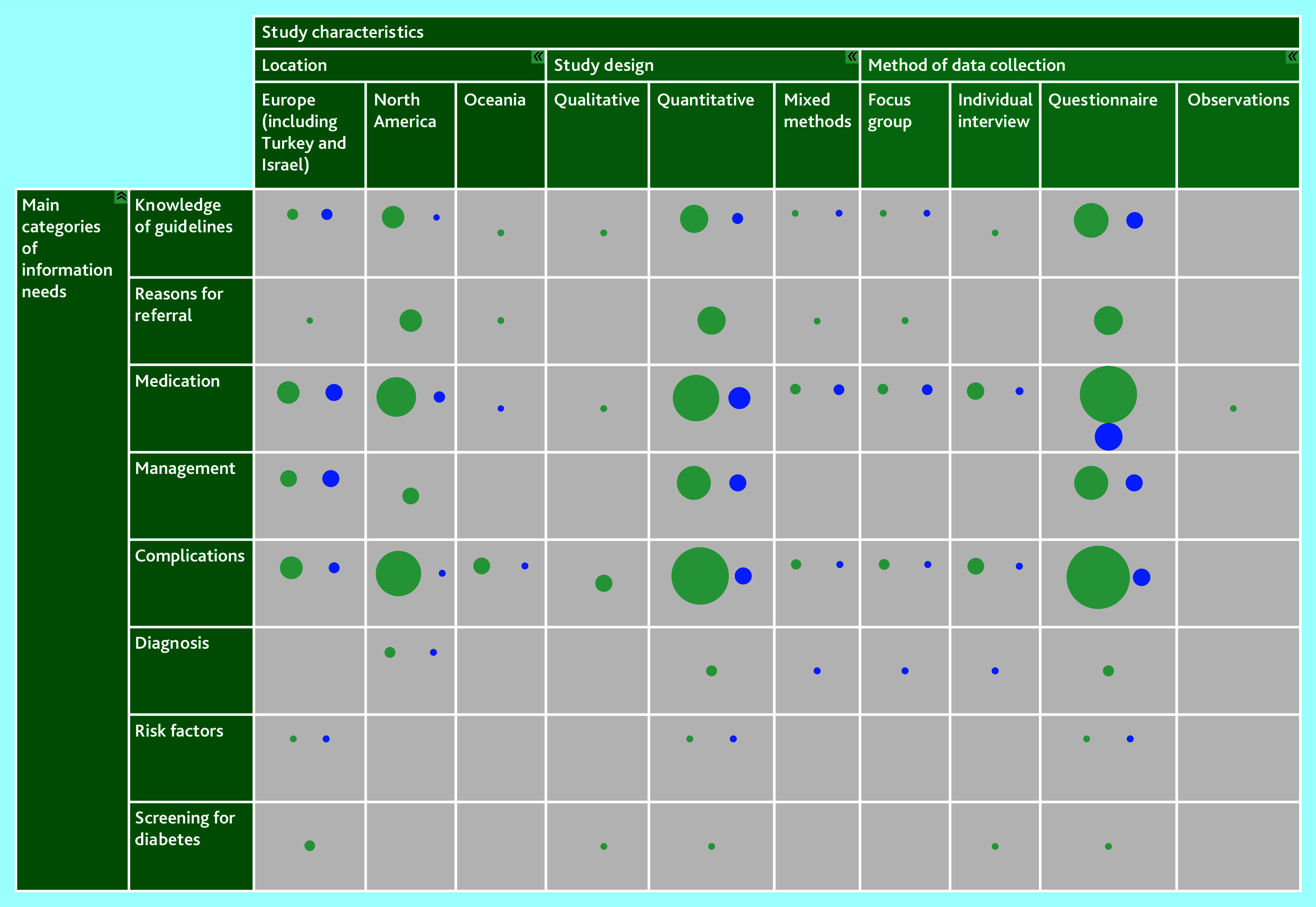
Evidence map. The interactive evidence map displays included studies as bubbles. The size of each bubble corresponds to the number of studies in the specific category it represents. Green bubbles indicate studies with a narrow focus, while blue bubbles represent studies with a broad focus. Interactive version available at: https://gapmap.danishdiabetesknowledgecenter.dk/informationneeds

The interactive evidence map (Figure 3; interactive version available at: https://gapmap.danishdiabetesknowledgecenter.dk/informationneeds) revealed apparent research gaps. No studies on management have been conducted in Oceania, no studies on diagnosis have been conducted outside North America, and no studies on risk factors or screening have been conducted outside Europe. Further, no qualitative data are available on information needs in the main categories management and risk factors.

## Discussion

### Summary

In a systematic review of 39 studies from Europe, North America, and Oceania, the information needs for GPs related to PWT2D pertained to medications, management, complications, referrals, risk factors, diagnosis, screening, and clinical guidelines. Twenty-nine included studies were quantitative, five were qualitative, and five were mixed methods; there were some concerns about risk of bias for most included studies.

Information needs were grouped into eight categories and 36 subcategories. Two general categories — knowledge of guidelines and reasons for referral — were related to all the remaining categories of medication*,* management, complications, risk factors, diagnosis, and screening.

### Strengths and limitations

To the best of the authors’ knowledge, this is the first systematic review of GPs’ information needs related to diabetes care. Another strength is the comprehensive search strategy and selection process, minimising the risk of missing relevant studies.

Heterogeneity in the included studies is present and both study type and outcomes are diverse, and interpretations should be considered carefully. Nonetheless, because in the current study the authors had knowledge about the literature within the field of research before commencing the research, the review methods were chosen to comprehend this. To develop a meaningful analysis, in the current study the authors chose to focus the present review on Western countries only. However, diversity in how primary care is organised in the included countries still proves a limitation to generalisability. The provided information needs model should be applied with local characteristics in mind.

In addition, four studies in Spanish, French, or Hebrew were eligible for inclusion based on titles and abstracts but not included (see Supplementary Information S2). The findings should be interpreted with due consideration of the descriptive and exploratory quality of included studies, which are not designed to demonstrate effect or causality.

### Comparison with existing literature

The categories of medication, management, and complications shed light on different aspects of caring for PWT2D, consistent with existing literature.^7^^,^^8^ However, the current review provides more specific insights into GPs’ information needs.

The scarcity of documented information needs related to diagnosis is surprising, given the focus on diagnosis in nearly every study synthesised by Daei *et al.*^8^ In addition, Del Fiol *et al* found that many of the physicians’ most frequent questions pertained to diagnosis.^7^ The few information needs related to diagnosis that were found could arise from the fact that, in comparison with other types of physicians, GPs serve as the entry point to the healthcare system and thus frequently diagnose patients with diabetes.

In contrast to previous studies, the current study did not identify information needs directly related to the prognosis of type 2 diabetes. The category of complications contains much of the information often considered as parts of the prognosis. However, in type 2 diabetes, this is better described by the multiple short- and long-term complications related to the condition, also emphasised in the guidelines.^2^^,^^67^^,^^68^ Further, the pharmacological management of hyperglycaemia has recently changed from concentrating only on glycaemic control management to focusing more broadly on patient satisfaction, quality of life, medication adherence, and overall health, depending on, for example, heart disease, kidney disease, or psychological illness.^69^

Only four included studies focused on risk factors and screening for type 2 diabetes. Only studies clearly linking the risk factor to type 2 diabetes were included, thus studies solely on known risk factors such as heart disease or pre-diabetes were not included. Nevertheless, these are independent and important categories, as GPs see many people at risk of type 2 diabetes.^70^

### Implications for practice and research

Treatment targets are extremely challenging to achieve for most patients with diabetes, and concerns about limited consultation time persist.^18^ In addition, lack of time and information-searching skills hinder physicians from exploring the literature,^7^^,^^8^ and limited resources are available in general practice to focus on a single disease among the many physicians must manage.^11^^,^^12^ Further, information overload, lack of trust in available information and knowledge about information sources, doubt about whether a usable answer exists, and simple forgetfulness are other barriers to physician information-seeking.^7^^,^^8^^,^^14^^,^^71^ Additionally, numerous websites from governmental institutions, private companies, and lay persons, accompanied by a growing amount of healthcare information distributed on social media, might be a growing barrier thus sustaining physician-to-physician knowledge sharing instead of inspiring them to search for information themselves.^72^

The current findings call for a readily available, widely disseminated, and easily usable guideline in electronic and printed forms. The findings can inform basic and continuing education for GPs, ensuring that needed information is available in digital and printed educational formats. However, to avoid information overload, GPs would benefit from the availability of a single website portal managed and developed by evidence-based healthcare specialists with no conflicting interests. Such a source should include, at minimum, relevant guidelines, indications for referral, and significant updates to type 2 diabetes management. Lastly, GPs should systematically develop their information-searching skills, which should also be highlighted as an essential skill throughout the professional education system.^73^

The complex management of type 2 diabetes, coupled with the limited consultation time for each person with diabetes in general practice, entails a dilemma for policymakers. There should be unambiguous guidelines and role descriptions that include clear steps for referring PWT2D to specialised care, and a population management strategy involving general practice, specialty care, and local health authorities could be initiated. However, this would necessitate significant changes to incentive structures in some countries.

Additional research is needed to address the gaps in the literature the authors of the current study have mapped, as well as to enhance the understanding of information needs.

In conclusion, GPs reported needs for information on a variety of topics related to type 2 diabetes. Higher-order needs for knowledge about clinical guidelines and indications for referral to specialised care overlapped with needs for specific content related to medications, management, complications, risk factors, diagnosis, and screening. The findings provide GPs, policymakers, and researchers with insight into potential educational needs in general practice settings in Western healthcare systems.

## Data

All data used in this study are publicly available.

## References

[1] International Diabetes Federation (IDF) (2021). IDF Diabetes Atlas, 10th edition.

[2] American Diabetes Association Professional Practice Committee (2022). Classification and diagnosis of diabetes: standards of medical care in diabetes — 2022. Diabetes Care.

[3] Davies MJ, D’Alessio DA, Fradkin J (2018). Management of hyperglycemia in type 2 diabetes, 2018. A consensus report by the American Diabetes Association (ADA) and the European Association for the Study of Diabetes (EASD). Diabetes Care.

[4] Kazemian P, Shebl FM, McCann N (2019). Evaluation of the cascade of diabetes care in the United States, 2005-2016. JAMA Intern Med.

[5] Egginton JS, Ridgeway JL, Shah ND (2012). Care management for type 2 diabetes in the United States: a systematic review and meta-analysis. BMC Health Serv Res.

[6] Irving G, Neves AL, Dambha-Miller H (2017). International variations in primary care physician consultation time: a systematic review of 67 countries. BMJ Open.

[7] Del Fiol G, Workman TE, Gorman PN (2014). Clinical questions raised by clinicians at the point of care: a systematic review. JAMA Intern Med.

[8] Daei A, Soleymani MR, Ashrafi-rizi H (2020). Clinical information seeking behavior of physicians: a systematic review. Int J Med Inform.

[9] Smith R (1996). What clinical information do doctors need?. BMJ.

[10] Williamson JW, German PS, Weiss R (1989). Health science information management and continuing education of physicians. A survey of U.S. primary care practitioners and their opinion leaders. Ann Intern Med.

[11] Pauker SG, Gorry GA, Kassirer JP, Schwartz WB (1976). Towards the simulation of clinical cognition. Taking the present illness by computer. Am J Med.

[12] Dawes M (2005). Critically appraised topics and evidence-based medicine journals. Singapore Med J.

[13] Clarke MA, Belden JL, Koopman RJ (2013). Information needs and information-seeking behaviour analysis of primary care physicians and nurses: a literature review. Health Info Libr J.

[14] Dawes M, Sampson U (2003). Knowledge management in clinical practice: a systematic review of information seeking behavior in physicians. Int J Med Inform.

[15] Isham A, Bettiol S, Hoang H, Crocombe L (2016). A systematic literature review of the information-seeking behavior of dentists in developed countries. J Dent Educ.

[16] Duran-Nelson A, Gladding S, Beattie J, Nixon LJ (2013). Should we Google it? Resource use by internal medicine residents for point-of-care clinical decision making. Acad Med.

[17] Davies K (2007). The information-seeking behaviour of doctors: a review of the evidence. Health Info Libr J.

[18] González-González AI, Dawes M, Sánchez-Mateos J (2007). Information needs and information-seeking behavior of primary care physicians. Ann Fam Med.

[19] Page MJ, McKenzie JE, Bossuyt PM (2021). The PRISMA 2020 statement: an updated guideline for reporting systematic reviews. BMJ.

[20] Rethlefsen M, Kirtley S, Waffenschmidt S (2021). PRISMA-S: an extension to the PRISMA statement for reporting literature searches in systematic reviews. Syst Rev.

[21] Lockwood C, Munn Z, Porritt K (2015). Qualitative research synthesis: methodological guidance for systematic reviewers utilizing meta-aggregation. J Evid Based Healthc.

[22] Slater L (2014). Product review: PubMed PubReMiner. J Can Health Libr Assoc.

[23] Haddaway NR, Grainger MJ, Gray CT (2021). citationchaser: an R package and Shiny app for forward and backward citations chasing in academic searching. https://github.com/nealhaddaway/citationchaser.

[24] Thomas J, Graziosi S, Brunton J (2023). EPPI-Reviewer: advanced software for systematic reviews, maps and evidence synthesis.

[25] Hong Q, Fàbregues S, Bartlett G (2018). The Mixed Methods Appraisal Tool (MMAT), version 2018 for information professionals and researchers. Education for Information.

[26] Booth A, Sutton A, Clowes M, Martyn-St James M (2022). Systematic approaches to a successful literature review.

[27] Digital Solution Foundry, EPPI-Centre (2022). EPPI-Mapper.

[28] Williamson JC, Glauser TA, Nevins PH (2013). Current practice patterns and identified educational needs of health care providers in managing patients with type 2 diabetes. Clin Diabet.

[29] Williamson C, Glauser TA, Burton BS (2014). Health care provider management of patients with type 2 diabetes mellitus: analysis of trends in attitudes and practices. Postgrad Med.

[30] Flory JH, Guelce D, Goytia C (2023). Prescriber uncertainty as opportunity to improve care of type 2 diabetes with chronic kidney disease: mixed methods study. J Gen Intern Med.

[31] de Lusignan S, McGovern A, Hinton W (2022). Barriers and facilitators to the initiation of injectable therapies for type 2 diabetes mellitus: a mixed methods study. Diabetes Ther.

[32] Chu L, Bhogal SK, Lin P (2022). AWAREness of diagnosis and treatment of chronic kidney disease in adults with type 2 diabetes (AWARE-CKD in T2D). Can J Diabetes.

[33] Marsden P, Grant J (1990). The learning needs in diabetes of general practitioners. Diabet Med.

[34] Phillips A, Dromgoole P (2005). Analysing training needs. Part 2: questionnaire results. J Diabetes Nurs.

[35] Shahla L, Vasudev R, Chitturi C (2017). Diabetes mellitus treatment – related medical knowledge among health care providers. Diabetes Metab Syndr.

[36] Thepwongsa I, Kirby C, Paul C, Piterman L (2014). Management of type 2 diabetes: Australian rural and remote general practitioners’ knowledge, attitudes, and practices. Rural Remote Health.

[37] Ates E, Set T, Saglam Z (2017). Insulin initiation status of primary care physicians in Turkey, barriers to insulin initiation and knowledge levels about insulin therapy: a multicenter cross-sectional study. Prim Care Diabetes.

[38] Cytryn KN, Garvey T, Hayes SM (2009). A qualitative assessment of educational opportunities for primary care providers in type 2 diabetes care. Diabetes Spectr.

[39] van Avendonk MJP, Gorter KJ, van den Donk M, Rutten GEHM (2009). Insulin therapy in type 2 diabetes is no longer a secondary care activity in the Netherlands. Prim Care Diabetes.

[40] Krall J, Gabbay R, Zickmund S (2015). Current perspectives on psychological insulin resistance: primary care provider and patient views. Diabetes Technol Ther.

[41] Rubin DJ, Moshang J, Jabbour SA (2007). Diabetes knowledge: are resident physicians and nurses adequately prepared to manage diabetes?. Endocr Pract.

[42] Gao Y, Peterson E, Pagidipati N (2020). Barriers to prescribing glucose-lowering therapies with cardiometabolic benefits. Am Heart J.

[43] Fogelman Y, Goldfracht M, Karkabi K (2015). Managing diabetes mellitus: a survey of attitudes and practices among family physicians. J Community Health.

[44] Wong T, Foote EF, Lefavour GS (1999). Physician knowledge and practice patterns relating to diabetic nephropathy. J Am Pharm Assoc (Wash).

[45] George J, Hannah S, Lang CC (2009). Thiazolidinediones and the influence of media adverse reporting on prescribing attitudes in practice (TZD-IMPACT) study. Cardiovasc Ther.

[46] Fisher SJ, Huang X, Pawaskar M (2018). Hypoglycemia in type 2 diabetes: understanding patients’ and physicians’ knowledge and experience. Endocrine.

[47] (2000). Attitudes of Italian physicians towards intensive metabolic control in type 2 diabetes. The QuED Study Group-Quality of Care and Outcomes in Type 2 Diabetes. Diabetes Nutr Metab.

[48] Ali M, Adams A, Hossain MA (2016). Primary care providers’ knowledge and practices of diabetes management during Ramadan. J Prim Care Community Health.

[49] Yilmaz T, Basara E, Yilmaz T (2021). Approaches and awareness of family physicians on diabetes management during Ramadan. Int J Clin Pract.

[50] Shubrook JH, Pak J, Dailey G (2020). Primary care physicians’ knowledge of the cardiovascular effects of diabetes medications: findings from an online survey. Adv Ther.

[51] Lo C, Teede H, Ilic D (2016). Identifying health service barriers in the management of co-morbid diabetes and chronic kidney disease in primary care: a mixed-methods exploration. Fam Pract.

[52] Delorme C, Boisjoly HM, Baillargeon L (1998). Screening for diabetic retinopathy. Do family physicians know the Canadian guidelines?. Can Fam Physician.

[53] Wiggins MN, Landes RD, Bhaleeya SD, Uwaydat SH (2015). Reprint of: Primary care physicians’ knowledge of the ophthalmic effects of diabetes. Can J Ophthalmol.

[54] Bissett SM, Stone KM, Rapley T, Preshaw PM (2013). An exploratory qualitative interview study about collaboration between medicine and dentistry in relation to diabetes management. BMJ Open.

[55] Poudel P, Griffiths R, Wong VW (2020). Perceptions and practices of general practitioners on providing oral health care to people with diabetes -a qualitative study. BMC Fam Pract.

[56] Garcia-Klepzig JL, Sanchez-Rios JP, Manu C (2018). Perception of diabetic foot ulcers among general practitioners in four European countries: knowledge, skills and urgency. J Wound Care.

[57] Lea JP, McClellan WM, Melcher C (2006). CKD risk factors reported by primary care physicians: do guidelines make a difference?. Am J Kidney Dis.

[58] Bilello J, Jupiter DC (2021). A pilot survey: knowledge of Charcot neuroarthropathy among family and internal medicine practitioners. J Foot Ankle Surg.

[59] Alabdali LAS, Jaeken J, Dinant G-J, Ottenheijm RPG (2019). Awareness of limited joint mobility in type 2 diabetes in general practice in the Netherlands: an online questionnaire survey. BMC Fam Pract.

[60] Gracen L, Hayward KL, Aikebuse M (2022). An exploration of barriers and facilitators to implementing a nonalcoholic fatty liver disease pathway for people with type 2 diabetes in primary care. Diabet Med.

[61] Özgüç H, Narmanli M, Çirnaz H (2021). Turkish primary care physicians’ attitudes and knowledge of obesity and bariatric surgery: a survey study. Turk J Surg.

[62] Rayanagoudar G, Moore M, Zamora J (2015). Postpartum care of women with gestational diabetes: survey of healthcare professionals.. Eur J Obstet Gynecol Reprod Biol.

[63] Whitford DL, Lamont SS, Crosland A (2003). Screening for type 2 diabetes: is it worthwhile? Views of general practitioners and practice nurses. Diabet Med.

[64] El-Beheiry M, Vergis A, Choi J-U (2020). A survey of primary care physician referral to bariatric surgery in Manitoba: access, perceptions and barriers. Ann Transl Med.

[65] Fürthauer J, Flamm M, Sönnichsen A (2013). Patient and physician related factors of adherence to evidence based guidelines in diabetes mellitus type 2, cardiovascular disease and prevention: a cross sectional study. BMC Fam Pract.

[66] Rätsep A, Kalda R, Oja I, Lember M (2006). Family doctors’ knowledge and self-reported care of type 2 diabetes patients in comparison to the clinical practice guideline: cross-sectional study. BMC Fam Pract.

[67] Cherney DZI, Repetto E, Wheeler DC (2020). Impact of cardio-renal-metabolic comorbidities on cardiovascular outcomes and mortality in type 2 diabetes mellitus. Am J Nephrol.

[68] Naicker K, Johnson JA, Skogen JC (2017). Type 2 diabetes and comorbid symptoms of depression and anxiety: longitudinal associations with mortality risk. Diabetes Care.

[69] Williams DM, Jones H, Stephens JW (2022). Personalized type 2 diabetes management: an update on recent advances and recommendations. Diabetes Metab Syndr Obes.

[70] Chatterjee S, Khunti K, Davies MJ (2017). Type 2 diabetes. Lancet.

[71] O’Connor M, O’Donovan B, Waller J (2020). Communicating about HPV in the context of head and neck cancer: a systematic review of quantitative studies. Patient Educ Couns.

[72] Klerings I, Weinhandl AS, Thaler KJ (2015). Information overload in healthcare: too much of a good thing?. Z Evid Fortbild Qual Gesundhwes.

[73] Löhönen J, Isohanni M, Nieminen P, Miettunen J (2009). A guide for medical information searches of bibliographic databases -psychiatric research as an example. Int J Circumpolar Health.

